# Qualitative evaluation of the outcomes of care and treatment for children and adolescents with nodding syndrome and other epilepsies in Uganda

**DOI:** 10.1186/s40249-019-0540-x

**Published:** 2019-04-30

**Authors:** Catherine Abbo, Amos Deogratius Mwaka, Bernard Toliva Opar, Richard Idro

**Affiliations:** 1 Department of Psychiatry, School of Medicine College of Health Sciences, Makerere University, Kampala, Uganda; 2 Department of Medicine, School of Medicine College of Health Sciences, Makerere University, Kampala, Uganda; 3 Ministry of Health, Kampala, Uganda; 4 Department of Paediatrics, School of Medicine College of Health Sciences, Makerere University, Kampala, Uganda

**Keywords:** Nodding syndrome, Epilepsy, Multi-disciplinary, Clinical audit, Clinical support supervision, Uganda

## Abstract

**Background:**

In 2012, the Ugandan Government declared an epidemic of Nodding Syndrome (NS) in the Northern districts of Gulu, Kitgum, Lamwo and Pader. Treatment guidelines were developed and NS treatment centres were established to provide symptomatic control and rehabilitation. However, a wide gap remained between the pre-defined care standards and the quality of routine care provided to those affected. This study is to qualitatively assess adherence to accepted clinical care standards for NS; identify gaps in the care of affected children and offer Clinical Support Supervision (CSS) to Primary Health Care (PHC) staff at the treatment centres; and identify psychosocial challenges faced by affected children and their caregivers.

**Methods:**

This case study was carried out in the districts of Gulu, Kitgum, Lamwo and Pader in Uganda from September to December in 2015. Employing the 5-stage approach of Clinical Audit, data were collected through direct observations and interviews with PHC providers working in public and private-not-for-profit health facilities, as well as with caregivers and political leaders. The qualitative data was analysed using Seidel model of data processing.

**Results:**

Clinical Audit and CSS revealed poor adherence to treatment guidelines. Many affected children had sub-optimal NS management resulting in poor seizure control and complications including severe burns. Root causes of these outcomes were frequent antiepileptic drugs stock outs, migration of health workers from their work stations and psychosocial issues. There was hardly any specialized multidisciplinary team (MDT) to provide for the complex rehabilitation needs of the patients and a task shifting model with inadequate support supervision was employed, leading to loss of skills learnt. Reported psychosocial and psychosexual issues associated with NS included early pregnancies, public display of sexual behaviours and child abuse.

**Conclusions:**

Despite involvement of relevant MDT members in the development of multidisciplinary NS guidelines, multidisciplinary care was not implemented in practice. There is urgent need to review the NS clinical guidelines. Quarterly CSS and consistent anticonvulsant medication are needed at health facilities in affected communities. Implementation of the existing policies and programs to deal with the psychosocial and psychosexual issues that affect children with NS and other chronic conditions is needed.

**Electronic supplementary material:**

The online version of this article (10.1186/s40249-019-0540-x) contains supplementary material, which is available to authorized users.

## Multilingual abstracts

Please see Additional file 1 for translations of the abstract into the five official working languages of the United Nations.

## Background

Nodding Syndrome (NS) is a neurological disorder with as yet undefined etiology. It affects children and adolescents aged 3–15 years and has mainly been observed in Sub-Saharan Africa. The disorder was first reported in 1960 in Tanzania, then in South Sudan in the 1990s [1] and subsequently in Uganda in the 2000s [2]. NS is a multi-system disorder that predominantly affects the central nervous, musculo-skeletal and endocrine systems [3] with key features including seizures, intellectual disability, and muscle wasting and growth retardation.

Literature indicates that areas with high prevalence of NS also have high prevalence of other forms of epilepsy [4], indicating possible shared etiological factors [5, 6]. In 1992, Ovuga and others reported the infection rate with *Onchocerca volvulus* in patients with epilepsy (61%) and retarded growth (70%) to be significantly higher than in the general population of Kyarusozi sub-county, Western Uganda [7]. Kaiser et al. (2009) referred to a phenomenon of head nodding observed in the Kabarole District in Western Uganda as possibly constituting a feature of an epileptic syndrome caused by *O. volvulus* [8]. Colebunders (2016) compared observations from three countries: South Sudan, Uganda and Democratic Republic of Congo and concluded that the *Simulium* (black) flies may play a key role in the transmission of an etiological agent that either directly or indirectly causes, not only NS, but also other forms of epilepsy in onchocerciasis endemic regions [4]. In 2012, The Ugandan Government declared an epidemic of NS in the Northern districts of Gulu, Kitgum, Lamwo and these regions also have a very high burden of other forms of epilepsy. The current guidelines recommend antiepileptic medications, nutritional rehabilitation, psychosocial intervention, nursing care and physical rehabilitation [9]. However little is known about how best to deliver and facilitate uptake of such interventions to improve care and outcomes for people with NS [10]. One way to enable effective clinical interventions is the development and adoption of clinical guidelines to support evidence-based practice [11].

In response to the NS epidemic, a multidisciplinary team was formed to develop clinical guidelines and set up specific treatment centres to offer care for children with NS. The guidelines and processes were published earlier [9, 12, 13]. A Clinical Audit (CA) conducted 1 year after introduction of the NS guidelines reported marked improvements in quantitative clinical and functional outcome measures [14]. However, subsequently the gap between the recommended standards of care for NS and the actual services delivered to NS patients has widened. The implementation of the national NS plan involved formation of the national and district task forces including the Gulu Regional Referral Hospital (GRRH) Task Force [12]. Established in 2013, one of the major mandates of GRRH NS Task Force, of interest to the CSS visits, was to support and provide leadership for the clinical care for the district health services in Gulu, Kitgum, Lamwo and Pader in the care of patients with NS through supervisory visits.

In this study, we report qualitative findings from annual CA and CSS 1 year after the initial quantitative audit and 2 years following the introduction of the NS clinical guidelines. “CA” describes the process of assessing clinical practice against accepted standards [9] and is internationally recognized as a key component of care quality assurance [14–17]; we qualitatively assessed adherence of NS clinical services providers to the accepted clinical guidelines. “Clinical Support Supervision” (CSS) is mandated by the Ugandan Ministry of Health (MoH) and describes the process of an experienced practitioner supporting less experienced colleagues, creating an environment in which the participants have an opportunity to evaluate, reflect and develop their practice [18, 19]. We used CSS with the aim of providing empathetic support to improve clinical and management skills for children with NS.

## Methods

### Study setting

This study was conducted in Northern Uganda between September and December 2015 where nodding syndrome has affected children in the districts of Gulu, Kitgum, Lamwo, Pader, Amuru and Lira. The total number of affected children in the above districts between January 2012 and March 2018 was 2143, with 128 deaths over the same period [20] (Table 1). There is also high prevalence of epilepsies registered in the health facilities in the NS-affected districts [21]. The populations in the four districts involved in this audit were: Gulu 275 613; Kitgum 204 048; Pader 178 004 and Lamwo 134 371, with a total of 792 736 [22]. The Acholi ethnic group is the most populous group in these districts. Notably, this region of Northern Uganda suffered a 20-year insurgency between the Lord’s Resistance Army (LRA) and the government of Uganda under the NRM rule during 1986 to 2006. This civil conflict forced the local population in Gulu, Kitgum and Pader districts into internally displaced person’s camps [23]. In addition, there is a high level of poverty in this region. The main source of livelihood is subsistence farming [22].

**Table 1:** Number of NS cases and deaths per district in northern Uganda

District	^a^Total Population 0–17 years (%)	^b^No. of Health facilities	^c^No. of NS Cases *n* (%)	^c^No. of Deaths due to NS No. (%)	^c^NS treatment/Rehabilitation centre
Pader	102 812 (58.2)	35	806 (38.7)	81 (62.8)	5
Kitgum	114 576 (56.5)	22	544 (26.1)	33 (25.6)	4
Lamwo	78 094 (58.2)	23	339 (16.3)	10 (7.8)	4
Gulu	142 111 (53.4)	73	323 (15.5)	1 (0.8)	1
Amuru	110 153 (59.5)	38	58 (2.7)	4 (3.1)	1
Lira	215 702(53.9)	29	13(0.6)	–	1

^a^National Population and Housing Census 2014, area specific profiles

^b^Ministry of Health Facilities Inventory, July 2012

^c^Minister of Health Statement of nodding syndrome in Northern Uganda March 2018

*NS* Nodding syndrome, − Not applicable

### Study design

Data were collected through direct observations and interviews with primary healthcare providers working in public and private-not-for-profit health facilities, as well as with political leaders of the four districts. Participants were sampled purposively from the following groups: GRRH NS Task Force, District Health Officers (DHOs), Caritas (a non-governmental organization) and caregivers of children with NS.

#### Audit of NS guideline implementation

CA of the MOH guidelines followed the 5-stage approach [24] with some modification to meet the needs of this particular audit [25]. These steps are: i) standard or guideline selection, ii) planning for the audit, iii) measuring performance, and iv) making and v) sustaining improvements. The standard used in the audit was the nationally endorsed clinical guidelines for management of NS [13]. In stage ii) we involved stakeholders including telephoning the DHOs and the GRRH Task Force team members that were available.

We used the Donabedian (1966) classification system of structure, process and outcome to determine areas of practice from which we selected our audit topics for emphasis [26]. For stage iii) we conducted in-person interviews with the groups listed above. District leaders helped with organizing meetings with parents and caregivers of children with NS.

#### Data analysis

We employed Seidel’s model (1998) of processing/analysis of data collected qualitatively by processes that are interlinked and cyclical [27]. We reviewed documentation including our stakeholder interview field notes, the recorded health facility observations and available written reports from the districts regarding NS management, to identify areas of adherence to or divergence from the NS clinical guidelines.

## Results

### Measuring performance

#### Quality of implementation

Planning meetings, initial setting up of the treatment centres and training of the primary healthcare workers were conducted in line with set standards. The MOH central CSS team found that the supervision team of GRRH completed the majority of their responsibilities including regular support supervisory visits, and in some cases, the provision of continuous professional education.

#### Gaps in service provision identified during CA

##### Suboptimal seizure management

The main gaps in the implementation of the national plan on NS were related to the management of seizures and seizure control:

i) *Adherence to treatment protocols:* There were many cases of failure to adhere to NS treatment protocols in the prescription of anti-seizure medications. For example, from the interviews, it was reported that many children with NS on a combination of two or more antiepileptic medication at the same time without indication. This corresponded to similarly suboptimal care for people with other epilepsies; for example many patients were found on fixed low doses of oral carbamazepine such as 200 mg twice daily and this dose was rarely adjusted for symptom control, weight gain or advancement in age.
ii) *Supply of medications:* The supply of anti-seizure medications and health unit stocks hardly lasted into the second month yet these were supplied quarterly. During these periods, the primary healthcare workers would rely on antiepileptic drugs and supplies obtained from the surrounding districts. Although oral phenytoin was available, a report of severe adverse events to phenytoin in two out of five patients in whom the primary healthcare workers had attempted to switch antiepileptic drugs discouraged them. The quotes below support the medication stock-outs:

*The problem is that sometimes even when you want to give the child the drugs like it is prescribed, you will go to the health facility and they tell you that the drugs are not there which creates a gap in the treatment of the children so what I think is that the drugs should be made available at the facility all the time* (A female caregiver, Health Facility 4).

*Sometimes you go there and they tell you the drugs are not there so you have to go and buy from the drug shops* (A female caregiver, Health Facility 3)*.*

*Sometimes, like currently some of the drugs that we give them is out of stock like the sodium valproate which normally helps them a lot, is out of stock and this is happening on many occasions where the facility runs out of the drugs* (A health worker, Health Facility 2).

iii) The Health workers reported very high patient load in one of the health facilities which is not adequately staffed to handle such high numbers of patients. This included many patients with epilepsies other than NS, who significantly increased the load of patients attending the clinic. Staff did not feel that they could turn these patients away

*Politics surrounding the disease aside and critical examination is done in these children you will realize that very few of these children actually have nodding syndrome, the majority of them have epilepsy instead but if you talk about that you may land into problems* (A male health worker, Health Facility 3).

##### Inadequate staffing

There was extreme shortage of human resources to manage NS in Health Facility 4, the main treatment centre in District 4. The staff numbers had reduced by 75% as some health workers had moved to other districts while others returned to higher education. There were only two Clinical Officers, including the in-charge who was also the NS focal person for the district and acting as District Health Education Officer. The only full-time nurse for the program in the health unit had a chronic medical condition which sometimes meant that he was unable to work. The work load, which in addition to care for NS patients included the usual primary healthcare (PHC) activities including vaccinations and home health visits, was overwhelming for the team.

Adding to this burden, patients from outside of Health Facility 4 catchment area also attended after Medical Teams International (MTI), previously caring for patients in two other health facilities, moved out of the district. Moreover, health workers employed to care for children with NS reported that many children with other epilepsies would also attend their facilities, further increasing their workload:

*You will realize that very few of these children actually have nodding syndrome, the majority of them have epilepsy instead but if you talk about that you may land into problems* (A male health worker, Health Facility 3).

The health workers were severely over-stretched and this often resulted in patients only having their medicines refilled rather than receiving a comprehensive review, or not receiving any treatment at all.

*Then for me the problem that I see is that sometimes the children come here but they find that there is no body to give them the drugs* (Male VHT, Health Facility 4).

Lack of adequate staffing placed an extreme burden on the remaining health workers, resulting in exhaustion and burnout, as one health worker described:

*Then also from the facility here, we are very few, when the disease had just started we were 16 but now we have remained only four and yet we see so many children in a day. You find that in a day I will see over three hundred children with Nodding Syndrome which I find that it affects the quality of service that I give because I get completely exhausted* (A male health worker, Health Facility 4).

Caregivers also expressed concerns that some health workers were inadequately trained to treat children with NS:

*There are some problems because what I have seen is that there were some health workers that were recruited as a response to this disease and they are not well-trained health workers so I see as if the way they respond to the patients who go to the health center to get the medicines is not good. Some people actually feel so disgusted and they leave the health facility without getting the medicines* (A female caregiver, Health Facility 4).

##### Gaps in rehabilitation services

Although the hospitals in the region had been improving their capacities in rehabilitation services, the services had been hampered by the absence of multidisciplinary rehabilitation specialists (speech and language therapists, occupational therapists, physiotherapists, nutritionists and mental health specialists) in the districts to sustain these services. For example, although GRRH had invested in surgical equipment for skin grafting and correction of contractures due to burns sustained during seizures, this service had not been implemented because the post-operative physical therapy that would be needed was not available in the districts. District 4 service staffing protocol had no positions for physiotherapists and occupational therapists in its establishment. Special needs schools and teachers are non-existent in this part of the country. The ordinary schools that are available are far off from where the children live, as one caregiver describes:

*The issue of schools, the children also travel very far from home to go to school up to Alune which you have seen even when you were coming here* (A male caregiver, Village 2).

#### Consequences for patients

Due to lack of access to anti-seizure medications and lack of access to adequately trained health workers, patients were off treatment for days and often for more than two months. Seizure control was often very poor. Consequently, complications of seizures were common, including frequent instances of severe burns due falling into cooking fire during a seizure. Many of these burns patients were admitted to health facilities and developed the further complications including life-threating infections and disabling contractures due to inadequate care.

*Yes, these children continue to suffer like that, there are others who fall in fire and they get burnt and deformed, sometimes the wounds are not treated and then also secondary infections come in and as a result they sometimes end up dying* (A female health worker, Health Facility 2).

#### Psychosocial issues

##### Men abandoning their families

Coming to terms with day to day challenges of having a child with NS is a complex process for the child, the caregivers and the wider community. We found that children affected with NS and their caregivers were greatly stigmatized in their communities. The stigma resulted into family separations, for example, with fathers in particular reported to be avoiding the home or leaving their marital homes to settle with another woman, as observed by this male Village Health Team (VHT) member:

*There are some of the parents who have left the issue of taking care of the children to the women because most of the men have now resorted to drinking so that they do not know what is happening in their homes, it is now mostly the women who are taking care of the children and most of the women have reached breaking point and some of the men even leave home and elope with other women to get peace of mind* (Male VHT, Village 3).

##### Child sexual abuse

Across all districts there were frequent reports of child sexual abuse and exploitation, with resultant pregnancies in multiple instances. One District Leader reported:

*…there are mainly defilement of Nodding cases and we are told of the 28 cases…. of the 28, there are fifteen pregnancies and the cases are before court. These are reported cases… We are suspicious that a number of cases are not being reported for example if someone comes that this son of mine has made a mistake and I have brought some little money you forgive him and this could be a very poor family… There is that tendency of receiving money from the perpetrators and the matter dies there. So that is why I am saying the unreported cases could even be more in the community* (A district leader, District 4).

Sexual relationships with girls under the age of consent are an illegal offence in Uganda and upon the reports of underage pregnancies in girls with NS the Ministers of Health and Gender gave a directive to immediately arrest the offending men involved in sexual exploitation of these girls [28]. One district politician reported that he had championed strengthening the criminal justice system in his district and that every case of defilement was handled without compromise:

*…Yeah, it is very common, these children are being abused sexually and there are a number of cases and ….Right now we have about four cases who are now serving prison sentences in District because of defiling these young girls. I think this is coming about because we have also customized and strengthened the issue of defilement in the district. I championed it starting on the 9th of October last year (2014) every case of defilement is handled well without compromise and it is working very well* (A district politician, District 4)*.*

However, there was some reticence from caregivers about reporting incidences of child sexual abuse to the police. There was a perception that police may be influenced by financial incentives that affected families would struggle to meet:

*If the police would establish its presence here it would be very good, but the bad thing is that even if you inform the police who are here in the sub-county they will still ask for money from you that they want fuel to take the culprit to----*(District 2)*, even coming here they will ask for fuel so the culprits are not afraid of doing these things* (A male caregiver, Village 2)*.*

Further, many parents and caretakers of girls with NS worried about who would marry their children and whether or not they would reproduce and reported feeling reassured when their girls became pregnant, whatever the circumstances of such pregnancies. One father of a 15-year-old confessed to one health worker that he wanted to try out and see if his daughter would get pregnant because he was worried of rejection from other men. He was in prison for impregnating his own daughter at the time of the CSS.

##### Sexual promiscuity in young people with NS

Additionally, there was a perception from parents that adolescents with NS had increased libido and were in fact voluntarily seeking sexual activity. Parents sometimes attributed their children’s promiscuous behaviour to the anti-seizure medications, in particular sodium valproate, even after the child had stopped taking the medication; this belief may be a contributing factor in poor compliance with treatment. Parents also expressed concern about the effects of anti-seizure medications on fetal development. Some respondents wondered whether these children and adolescents could be availed with contraception.

##### Other forms of child abuse

There were reports of children being exploited for labour, for example, a Bishop from a Pentecostal church who had taken on some children with NS, claiming to be praying for their healing while they lived in his church, was reported to use the children as labourers to cultivate his gardens.

##### Maltreatment of caregivers

The burden of care for children with chronic illness in our setting often falls on mothers. Those with children with NS described a sense of being let down by their spouses who are not providing the support expected of them. Mothers reported that when they asked their partners for support with childcare this sometimes resulted in their partners becoming abusive. This situation was described by a district leader:

*At family level, I think a lot is done by women, the care majorly is handled by the women; the men are not very committed to the care of these children as usual in our culture they have also extended it to the children. Yes it is also causing the problems of domestic violence in the families, in the care of these children, there is a gap because the women always complain that when the men go out they do not come with something to support the family and this normally creates a crack in the family and there have been cases of domestic violence in the families because of that* (A district leader, District 4).

##### Caregiver emotional distress and mental health

Chronic disease-related daily activities have been found to be a risk factor for emotional distress and poor adjustment, particularly in mothers [29]. We similarly found frequent reports of low mood and emotional distress in the mothers of children with NS [29]:

*These families are depressed, we have a mother who came in yesterday… for the last 1 week when we have shortage of drugs the daughter has been fitting* (having frequent seizures) *and yesterday her* (the mother’s) *eyes were swollen from crying, she was crying day and night… because she is very poor… and now the daughter who is 16 years old is in that condition* (A male health worker, Health Facility 1).

### Identifying reasons why standard care was not met

**Fig. 1:**
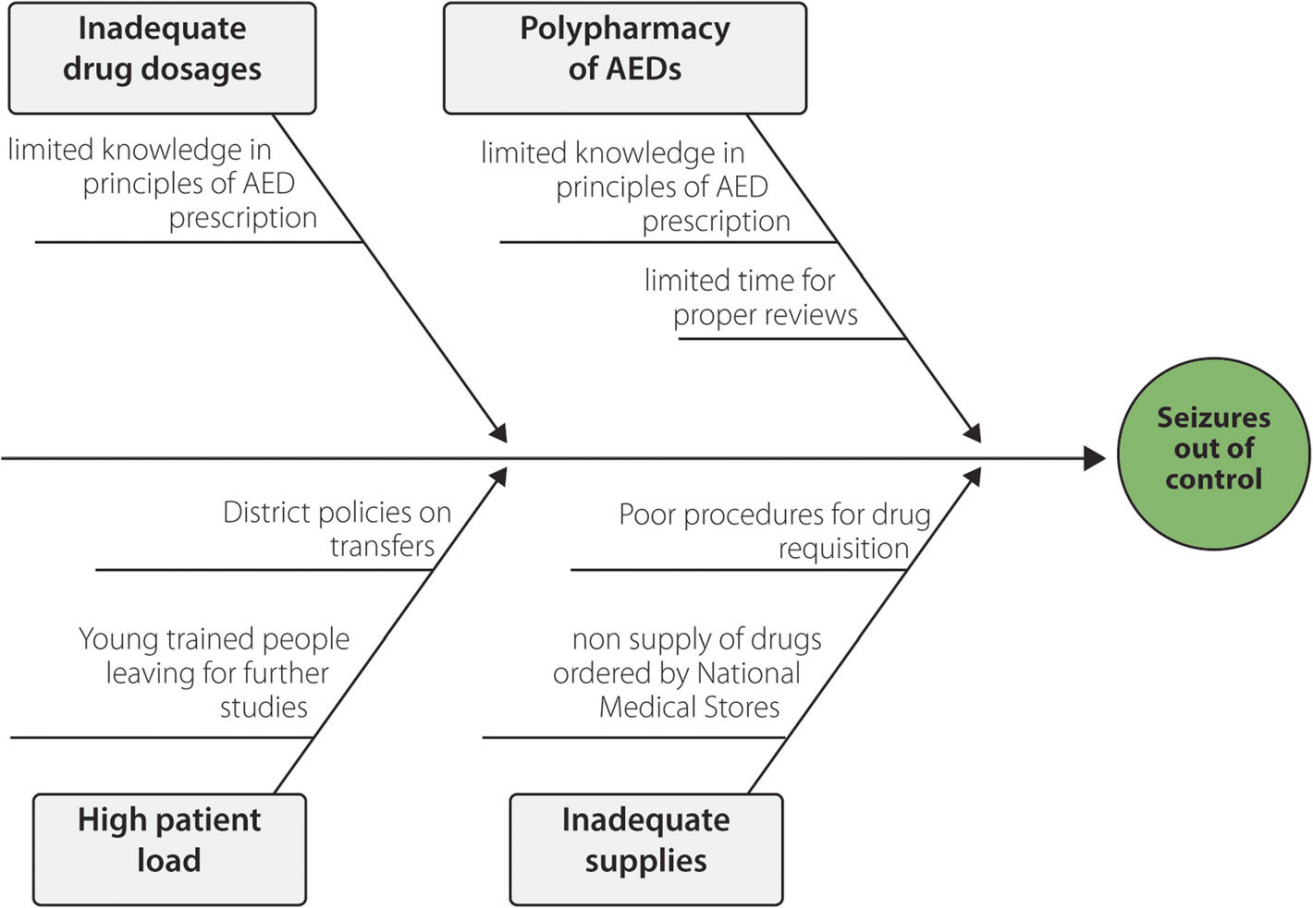
Fishbone (Ishikawa) diagram to understand possible root causes for seizures getting out of control. Legend: We first identified the four main causes using 3Ps i.e. People, Policies and Procedure (the big arrows). Next, we brainstormed about the causes of the main causes (the small arrows). AED: Anti-Epileptic Drugs

To identify some of the reasons why the NS guidelines or standard were not met for seizure control, we employed the fishbone diagram (Fig. 1).

#### Local political climate

In Uganda health service provision has been decentralized to the district level with the aim of devolving decision-making autonomy and promoting local ownership by the populations in receipt of these services. Local politicians therefore have the power to dictate local health policy. Some interviewees reported that NS is politicized within the political sphere with negative repercussions for those affected, as this health worker described:

*There is one thing, which is very serious with Nodding Syndrome that is the issue of politicians politicizing this disease so much to the extent that it is almost illegal to talk about this disease. If you ask someone about nodding syndrome there in the community he may shy away, not that he does not have information but because of that political aspect they fear to talk, you know people are using this nodding disease for very many reasons, myself, I do not want to associate myself with nodding syndrome because I know the implications. I treat them alright but I do not get involved in the discussions and things like that because the politicians have taken over the whole thing even more than the health worker* (A male health worker, Health Facility 3)*.*

### Making improvements: tentative approaches to fill the gaps

#### Need for better training and guidelines for epilepsy management

The CA and CSS team noted major challenges with patient care. Although, in general the health and quality of life of patients in all the districts improved in the first year of implementation of the NS guidelines [14], there remains major gaps in the quality of care provided. We identified a high incidence of non-adherence to guidelines for anti-seizure medication prescribing, for example the prescription of multiple drugs at low dose rather than first trying maximum dose of a single agent to achieve seizure control. Incorrect prescribing practices were partly addressed through peer counselling and in formal Continuous Medical Education (CMEs) in the districts and later, specifically tailored trainings for the treatment of NS and the principles of treatment of epilepsy. Given that health workers caring for NS patients also care for many people with other epilepsies, there is urgent need to revise the NS guidelines to include recognition and management of other common epilepsies, and to train health workers on the revised guidance.

The Ministry of Health needed to conduct refresher training in the management of NS and in the principles of epilepsy care.

The outreach programs, which stalled should be restarted urgently. The District Health Officer (DHO) and Chief Administrative Officers (CAOs) were requested to deal with the transport issues and get their district vehicles running.

#### Planning for consistent anti-seizure drug supplies

The CA and CSS team supported plans to quantify the exact requirements of antiepileptic drugs, especially in District 4, to prevent stock shortages. A census of patients with NS and other epilepsies was planned in order to estimate the treatment burden. Written guidance is also needed for when there is national shortage of specific drugs; for example, when carbamazepine is lacking, phenytoin could be used as a substitute and initiated slowly. Sodium valproate, which is the first line recommended treatment for seizures in NS, should be included on the National Essential Drugs List.

#### Adequate staffing and patient flow management

Severe health worker shortages resulted in unmanageable workloads for remaining staff. We recommended that Health Facility 4, with an extremely high caseload of patients, be considered for upgrade to a Health Centre IV to enable adequate funding and staffing to deal with the growing patient load. Further work is needed to explore the reasons why patients seek care at particular health facilities for which they may be out of catchment, rather than seeking support more locally.

#### Multi-disciplinary rehabilitation services

We identified a severe lack of rehabilitative services across districts, yet the need for such services among the NS population was high. In District 4, there was no funding allocated to employ therapists involved in rehabilitation; whilst in another district, two physiotherapists and one occupational therapists had left and not been replaced. The study team recommended to participating districts that they should consider funding rehabilitation services. Alternatively, Gulu Regional Referral Hospital, the current base for the NS task force, could be supported to provide the required services and patients referred to this centre from district health facilities.

#### Burns prevention and management

Specifically addressing management of severe burns, the discussion with GRRH team included suggestions of peer mentorship and burns management training by the Plastic Surgery team from the Mulago National Referral Hospital in Kampala.

Additionally, a region-wide Community Education program for burns prevention should be initiated. This was discussed with the DHOs and Caritas-Uganda a non-governmental organization supporting community based programs in all the participating districts, who have agreed to support such an initiative. We recommended that Caritas build burns prevention education into existing community support programs, for example advising families on safe fireplace construction to reduce the risk of a person falling into the fire during an epileptic seizure.

#### Psychosocial support for NS patients and their families

In supporting health workers and families to cope with the wide-ranging psychosocial issues affecting NS patients and their caregivers, the Study Team recommended adopting the models of psychosocial support from Caritas and involving the community development officers whose major role is to improve the quality of life of community members. Additional support, for example with food provision, was needed for families with multiple affected persons. In the two sub-counties where Caritas is working, some of these issues were partially being addressed via parent-to-parent contacts.

In one meeting in one of the districts, there was a suggestion that separate guidelines should be developed to guide the management of psychosocial issues related to nodding syndrome followed by training of health workers to help community development officers better manage some of the psychosocial issues identified. It may be beneficial to seek the support of local churches, mosques and traditional healers in developing support structures for families affected by NS.

Regarding specific cases of potential child exploitation identified through our interviews, we recommended that the relevant district leaders follow up these matters initially to fully understand the facts, before pursuing further action, including legal intervention, as appropriate. For example, in the case of the bishop reported to be using children with NS for unpaid labour, it was felt that he may also be providing psychosocial support and respite for the affected families, therefore it might be possible for him to stop exploiting the children for labour but to continue to provide much-needed pastoral care.

#### Research into the causes of NS to facilitate better treatment in future

Since 2009, the MOH, WHO, the US CDC and other partners have conducted epidemiological studies that yielded the information currently known about the disease [2, 3]. These investigations enabled exclusion of a number of possible infectious, genetic, toxic or nutritional factors, but the definite cause of NS remains unknown. Onchocerciasis is deemed the most likely etiologic agent in NS [30, 31]. A deficiency of vitamin B6, A, Selenium and Zinc was found in most affected children, but this same deficiencies were also found in children without NS living in the same area [32]. The current ongoing randomized trial “Doxycycline for the Treatment of Nodding Syndrome Trial” may give more information on the etiology of NS [33].

#### Continued collaborations

The Office of the Prime Minister (OPM) of Uganda led and coordinated a multidisciplinary response to NS, with the aspects of management pertaining to healthcare handled by the MOH; nutrition by Ministry of Disaster Preparedness (MDP); hygiene and sanitation by Ministry of Water and Environment; and finance by Ministry of Finance (MOF). Other sectors and partners were also identified and requested to deal with the situation and conditions within their mandate and areas of competence [12]. These collaborations worked very well in the beginning judging from improved patient care standards resulting in improvements in seizure control and other patient outcomes. However, our CA and CSS findings indicated that over time, these collaborations weakened, with some partners reducing the level of support provided or withdrawing altogether. We recommend returning to the initial working model of specific Ministries having dedicated responsibility for particular aspects of NS management, working in collaboration to improve the quality care for children with NS.

## Discussion

In the first year of implementation of the national NS response plan, we observed improvements in global functioning and good clinical seizure control in children with NS. This was probably a result of the training and enthusiasm of clinical care providers when the program was set up. However, this was not sustained and subsequent CA and CSS visits revealed that a high number of children experienced break-through seizures and frequent complications including burns injuries, due to incorrect anti-seizure medication prescriptions, poor medication adherence and lack of drug availability. Wide-ranging psychosocial issues further contributed both to poor seizure control and to high financial and emotional burden on families caring for children with NS.

The Budget Monitoring and Accountability Unit (BMAU) of Ugandan Ministry of Finance and Economic Planning reports have continually highlighted the issue of medical stock-outs at various levels. In Financial Year (FY) 2009/10 only 21% of the health facilities reported no stock-outs of tracer medicines (e.g. antimalarial) in the previous 6 months [34]; this may be worse for less frequently prescribed [34] medicines including antiepileptic drugs, in particular those such as Sodium Valproate which is not yet on the National Essential Drugs List despite being the guideline-recommended first line treatment for NS and being included on the World Health Organization (WHO) Essential Medicines List. Some of the reasons for drug stock out in Uganda health facilities reported by BMAU include poor planning, prioritization and forecasting; inadequate data management and monitoring systems to track amounts of drugs ordered, dispensed, prescribed and balances; non-supply of ordered items by National Medical Stores (NMS); and polypharmacy tendencies characterized by some clinicians prescribing more drugs for patients than needed. In our CA and CSS, non-supply of ordered items by NMS and polypharmacy tendencies were the major reasons for drug stock out and consequent poor seizures control. These factors will need to be addressed through better understanding of the local NS and epilepsy burden to inform appropriate drug ordering, and by improved health worker training to promote adherence to prescribing guidelines.

In the NS guidelines, Sodium Valproate is the first line drug of choice. Families expressed the belief that this caused ‘hypersexual’ behaviour in adolescents with NS; we found that caregivers maintained this belief even when their children exhibited the same behaviour when not taking the drug. Adolescents, including those not taking medication, were reported to exhibit sexualized behaviours in public, including masturbation. Children with NS develop intellectual disability, including limited understanding of social norms, therefore we feel that the reported inappropriate public sexual behaviour is due to intellectual disability rather than abnormally “hyper-sexualized” behaviour. Sodium Valproate’s side effects on sexual behaviour have not been sufficiently studied [35], but the available literature suggests that hypo- rather than hyper-sexuality may be a consequence of taking sodium valproate [35–37]. There is evidence that the use of valproate by females with bipolar affective disorders and epilepsy may cause menstrual cycle abnormalities, polycystic ovary syndrome, and hyper-androgenism [35], whilst in males a degree of sexual dysfunction has been reported but this may be associated with focal-onset [35] temporo-limbic seizures rather than with the medication [38]. It is therefore unlikely that sexual behaviours seen in NS adolescents are as a result of Sodium Valproate. Ongoing psychosocial support interventions will need to include education for families and continued social skills teaching for adolescents who publically display sexual behaviour.

The psychosocial and psychosexual challenges we identified need to be interpreted and discussed in the general context of Northern Uganda. Child sexual abuse and exploitation and associated teenage pregnancies, as well as other forms of child abuse, are not unique to the population of children with NS. The Uganda Bureau of Statistics report of 2016 indicated that Northern Uganda has the highest levels of poverty in the country, with 23.9% classified as chronically poor [22]. The majority of household heads have no formal education [22]. The region also has the highest teenage pregnancy rate at 64% and early marriages at 59% compared to the rest of Uganda at 24% [39]. A brief survey of mothers of adolescents with NS showed that nearly all of those surveyed had had their first pregnancy before the age of 18 years. Interventions to reverse this trend therefore have to be contextualized and should include all children and adolescents of both sexes; not just those with NS. It is possible that isolating children and families affected with NS and targeting them only for psychosocial interventions like provision of food and health care while leaving out the others might have contributed to the decline in improvement, if this resulted in social alienation of such families. It is, however, important to note that Uganda has a number of policies in place regarding child protection and prohibition of child labour and abuses. Uganda has also ratified a number of internationally-supported instruments to protect the rights of children including child sexual abuse and early marriage, including the National Strategy on ending child marriage and teenage pregnancy that was launched in 2017. These policies must be legally enforced, notwithstanding barriers to legal recourse reported by the families surveyed, such as police demanding financial incentives.

We found that the majority of fathers of children with NS distanced themselves from the care of their children with NS. It was not clear from this CA and CSS whether the apparent neglect by fathers in the care of children with NS is a coping strategy for the stressful experience of having a child with NS, or a cultural issue; further study is needed to explore these issues. Some studies have reported that coping strategies that involve distancing, escape and avoidance are more likely to be used when high levels of stress are experienced by parents [40]. Fathers are reported to be more likely to distance themselves from stressful family situations and may choose to work more in other aspects of life, avoid clinic visits or even ignore the condition. It has been argued that fathers use distancing when they experience their partners as being too involved, and therefore feel rejected and give up [41]. Ultimately, mothers are often left with all the responsibilities of caring for their children with NS, causing these mothers high levels of psychological distress. Support interventions for both parents, including counselling, household income and nutrition support and improved clinical care and seizure control in affected children, could improve the capacity of whole families to care for their children with NS and in particular help mothers who are differentially overburdened as care providers.

Finally, improvement in care for those with NS demands improved health service provision at all levels. In particular staffing numbers and skills mix need to be addressed at both national and district levels, in line with the recommendations set out in the NS guidelines. We found that loss of skills learnt in training due to inadequate supervision, replacement of trained staff with clinical staff not trained in NS care, and overall attrition of staff without replacement, were important findings relating to inadequate care for the children with NS. In addition, whilst a broad multidisciplinary team including members from the national NS Center at Gulu Regional Referral Hospital led the development of multidisciplinary NS clinical guidelines, implementation of these guidelines was poor, largely due to lack of staff available to facilitate the recommended practices.

Functional multidisciplinary teams will be key to improving holistic care for children with NS and their families. Inter-disciplinary rehabilitation is considered as the gold standard when considering patient outcomes regardless of the populations being studied [42]. Most high-income countries employ Multi-Disciplinary Teams (MDT) in the care of children with epilepsy and disabilities, however these are lacking in many low and middle income countries including Uganda. The situation is worse in poorly staffed rural areas. Although the initial MDT that developed the NS guidelines included occupational therapists, speech therapists, nutritionists, physiotherapists, neurologists, physicians and psychiatrists, these professionals are not available at the majority of health facilities treating children with NS [13]. In consequence we observed that facilities would use task-shifting to delegate tasks that would be performed by dedicated therapists to healthcare workers with lower qualifications [43, 44]. Task-shifting as a strategy is endorsed by the WHO, with emphasis on the need for tasks to be carefully selected, roles well defined and adequate supervision put in place [19, 45], however in our study we observed that supervision was not adequate. Studies have shown that training in skills without follow up support supervision in the next 3 months quickly results in loss of skills learnt [19, 46, 47]. Therefore whilst we will continue to support task shifting in health facilities caring for children with NS, with support from the MDT at Gulu Regional Referral Hospital, we recommend quarterly support supervisions to maintain the skills learnt and regular training including in any guideline updates, to ensure optimal care of children with NS. Urgent review of the clinical guidelines to reflect the current needs of children and adolescents with NS is required. As part of this study, a rehabilitation strategy needs to be developed and implemented in the NS affected districts. A theory of change behaviour could be used to guide strategic planning and to identify the current situation (in terms of needs and opportunities), the intended situation and what needs to be done to move from one to the other. This can help to design more realistic goals, clarify accountability and establish a common understanding of the strategies to be used to achieve the goals [48]. A theory of change explains how activities are understood to contribute to a series of results that produce the final intended impacts to the satisfaction of those who will use it [48]. Children with NS have multidisciplinary needs that call for better integrated care across health, education and social sectors.

### Limitations of this case study

Generalization of findings from this study is limited by the case study design. However, a case study strategy was the most appropriate for this CA and CSS. In this study we present qualitative data, whereas the one-year audit after initial implementation of the NS guideline reported quantitative results. Nonetheless, we believe that our current qualitative study is replicable, gives invaluable insights into the reasons for difficulties in realizing the recommended standards of care for NS and will support important technical recommendations to the MoH to facilitate future improvements.

## Conclusions

The first year of implementation of the NS national response plan was considered successful as evidenced by the noted improvements in both global functioning of the affected children, and clinically by reduction in seizure frequency. However, these improvements were not sustained and subsequent CA and CSS visits revealed that a number of children had break-through seizures and sustained burns largely as complications of poor management of seizures with antiepileptic drugs and frequent medicines stock-outs. The lack of a multi-disciplinary team at the implementation level in the districts could explain in part the failure in sustainability of high quality care after the national team who trained primary healthcare workers and helped in establishment of the NS treatment centres left the districts. Task-shifting could not at present adequately substitute the need for a multidisciplinary team for management of NS. Training and appropriate distribution of primary healthcare workers are important for the management of NS.

Psychosocial challenges associated with NS including inappropriate public sexual displays and advances, sexual abuse with accompanying teenage pregnancies, and various other forms of child abuse were common though not unique to children affected with NS. Proper implementation of existing Ugandan policies for the protection of children’s rights will be an important step. Targeted interventions to address the complex psychosocial issues we identified will require involvement of multiple stakeholders and should be holistic for the society and not limited to the NS affected children and families. To achieve this, the initial collaboration that worked well needs to be reinstated.

## Additional file

Additional file 1: Multilingual abstracts in the five official working languages of the United Nations (PDF 374 kb)

## Abbreviations

CA: Clinical audit
CDC: Centre for Disease Control
CSS: Clinical support supervision
HC: Health centre
LMIC: Low and middle income country
MDT: Multidisciplinary team
MOH: Ministry of Health
NS: Nodding syndrome
UBOS: Uganda Bureau of Statistics
WHO: World Health Organization

## References

[1] Spencer PS, Vandemaele K, Richer M, Palmer V, Chungong S, Anker M (2013). Nodding syndrome in Mundri county, South Sudan: environmental, nutritional and infectious factors. Afr Health Sci.

[2] Foltz JL, Makumbi I, Sejvar JJ, Malimbo M, Ndyomugyenyi R, Atai-Omoruto AD (2013). An epidemiologic investigation of potential risk factors for nodding syndrome in Kitgum District, Uganda. PLoS One.

[3] Idro Richard, Opoka Robert Opika, Aanyu Hellen T, Kakooza-Mwesige Angelina, Piloya-Were Theresa, Namusoke Hanifa, Musoke Sarah Bonita, Nalugya Joyce, Bangirana Paul, Mwaka Amos Deogratius, White Steven, Chong Kling, Atai-Omoruto Anne D, Mworozi Edison, Nankunda Jolly, Kiguli Sarah, Aceng Jane Ruth, Tumwine James K (2013). Nodding syndrome in Ugandan children—clinical features, brain imaging and complications: a case series. BMJ Open.

[4] Colebunders R, Hendy A, Mokili JL, Wamala JF, Kaducu J, Kur L (2016). Nodding syndrome and epilepsy in onchocerciasis endemic regions: comparing preliminary observations from South Sudan and the Democratic Republic of the Congo with data from Uganda. BMC Res Notes.

[5] Kaiser C, Asaba G, Leichsenring M, Kabagambe G (1998). High incidence of epilepsy related to onchocerciasis in West Uganda. Epilepsy Res.

[6] Kaiser C, Kipp W, Asaba G, Mugisa C, Kabagambe G, Rating D (1996). The prevalence of epilepsy follows the distribution of onchocerciasis in a west Ugandan focus. Bull World Health Organ.

[7] Ovuga E, Kipp W, Mungherera M, Kasoro S. Epilepsy and retarded growth in a hyperendemic focus of onchocerciasis in rural western Uganda. East Afr Med J. 1992;69:554–6. 1473507

[8] Kaiser C, Pion S, Boussinesq M (2009). Head nodding syndrome and river blindness: a parasitologic perspective. Epilepsia..

[9] Idro R, Musubire KA, Byamah Mutamba B, Namusoke H, Muron J, Abbo C (2013). Proposed guidelines for the management of nodding syndrome. Afr Health Sci.

[10] Buttery Y, Bury TaM J (1998). Implementing evidence through audit. Evidence-based healthcare: a practical guide for therapists.

[11] Grol R (2001). Successes and failures in the implementation of evidence-based guidelines for clinical practice. Med Care.

[12] Mwaka D, Kitara D, Orach C (2015). The enigmatic nodding syndrome outbreak in northern Uganda: an analysis of the disease burden and national response strategies. Health Policy Plan.

[13] Musisi S, Akena D, Nakimuli-Mpungu E, Abbo C, Okello J (2013). Neuropsychiatric perspectives on nodding syndrome in northern Uganda: a case series study and a review of the literature. Afr Health Sci.

[14] Idro R, Namusoke H, Abbo C, Mutamba BB, Kakooza-Mwesige A, Opoka RO (2014). Patients with nodding syndrome in Uganda improve with symptomatic treatment: a cross-sectional study. BMJ Open.

[15] Madden D (2008). Building a culture of patient safety. Report of the commission on patient safety and quality assurance.

[16] Esposito P, Dal Canton A (2014). Clinical audit, a valuable tool to improve quality of care. General methodology and applications in nephrology. World J Nephrol.

[17] Burgess R. New principles of best practice in clinical audit. London: Radcliffe Publishing; 2011.

[18] Wright SG, Elliott M, Scholefield H (1997). A networking approach to clinical supervision: clinical supervision offers support in clinical and professional development. Nurs Stand.

[19] Kilminster S, Jolly B (2000). Effective supervision in clinical practice settings: a literature review. Med Edu.

[20] Acheng JR. Statement on nodding sundrome in Northern Uganda. Kampala: Ugandan Ministry of Health; 2018. https://www.google.com/search?biw=1366&bih=625&ei=eYK5XMmNKsW21fAPmcyn-AU&q=statement+in+nodding+syndrome+in+uganda&oq=statement+in+nodding+syndrome+in+uganda&gs_l=psy-ab.3..33i22i29i30.674057.685211..685991...1.0..4.429.13291.2-24j17j2......0....1..gws-wiz.....6..0i71j35i39j0i131j0j0i67j0i10j0i22i30j0i13i5i30j33i160.96RgQinBvaQ. Acessed 17 Oct 2018.

[21] Duggan M (2013). Epilepsy and its effects on children and families in rural Uganda. Afr Health Sci.

[22] Uganda Bureau Of Statistics (2016). Demographic and health survey, statistical abstract.

[23] Branch A (2009). Humanitarianism, violence, and the camp in northern Uganda. Civil Wars.

[24] Foy R, Eccles MP, Jamtvedt G, Young J, Grimshaw JM, Baker R (2005). What do we know about how to do audit and feedback? Pitfalls in applying evidence from a systematic review. BMC Health Serv Res.

[25] Lokuarachchi S (2006). Clinical audit-what is it and how to do it?. Galle Med J.

[26] Donabedian A (1966). Evaluating the quality of medical care. Milbank Mem Fund Q.

[27] Seidel J, Seidelb J (1998). Qualitative Data Analyisis. The Ethnograph v5.0 A user’s guide (Appendix E).

[28] Otto A (2012). Minister warns against exploiting nodding-girls. The Observer News Paper.

[29] Cadman D, Rosenbaum P, Boyle M, Offord DR (1991). Children with chronic illness: family and parent demographic characteristics and psychosocial adjustment. J Pediatr.

[30] Colebunders R, Carter YJ, Olore PC, Puok K, Bhattacharyya S, Menon S (2018). High prevalence of onchocerciasis-associated epilepsy in villages in Maridi County, republic of South Sudan: a community-based survey. Seizure.

[31] Mukendi D, Tepage F, Akonda I, Siewe JNF, Rotsaert A, Ndibmun CN (2019). High prevalence of epilepsy in an onchocerciasis endemic health zone in the Democratic Republic of the Congo, despite 14 years of community-directed treatment with ivermectin: a mixed-method assessment. Int J Infect Dis.

[32] Spencer PS, Mazumder R, Palmer VS, Lasarev MR, Stadnik RC, King P (2016). Environmental, dietary and case-control study of nodding syndrome in Uganda: a post-measles brain disorder triggered by malnutrition?. J Neurol Sci.

[33] Anguzu R, Akun PR, Ogwang R, Shour AR, Sekibira R, Ningwa A (2018). Setting up a clinical trial for a novel disease: a case study of the doxycycline for the treatment of nodding syndrome trial–challenges, enablers and lessons learned. Glob Health Action.

[34] Minsitry of Finance and Economic Planning, Budget Monitoring and Accountability Unit (BMAU): Continuous stock-outs of medical supplies in Uganda: what are the root causes? BMAU Briefing Paper 2015. https://www.finance.go.ug/mofped/budget-monitoring-and-accountability-unit. Acessed 13 Feb 2019.

[35] Aldemir E, Akdeniz F (2009). Effects of valproate on male reproductive functions. Turk Psikiyatr Derg.

[36] Herzog A, Drislane F, Schomer D, Pennell P, Bromfield E, Dworetzky B (2005). Differential effects of antiepileptic drugs on sexual function and hormones in men with epilepsy. Neurology..

[37] Herzog AG, Drislane FW, Schomer DL, Pennell PB, Bromfield EB, Kelly KM (2004). Differential effects of antiepileptic drugs on sexual function and reproductive hormones in men with epilepsy: interim analysis of a comparison between lamotrigine and enzyme-inducing antiepileptic drugs. Epilepsia..

[38] Herzog AG (2008). Disorders of reproduction in patients with epilepsy: primary neurological mechanisms. Seizure.

[39] United Nations International Children’s Emergency Fund (UNICEF) (2017). The National Strategy to end early marriages and teenage pregnancy 2014/2015–2019/2020.

[40] Cousino MK, Hazen RA (2013). Parenting stress among caregivers of children with chronic illness: a systematic review. J Pediatr Psychol.

[41] Mitchell SJ, Hilliard ME, Mednick L, Henderson C, Cogen FR, Streis R (2009). Stress among fathers of young children with type 1 diabetes. Fam Syst Health.

[42] Ennion L, Rhoda A (2016). Roles and challenges of the multidisciplinary team involved in prosthetic rehabilitation, in a rural district in South Africa. J Multidiscip Healthc.

[43] Chen L, Evans D, Evans T, Sadana R, Stilwell B, Travis P (2006). Working together for health. The World Health Report.

[44] Dovlo D (2004). Using mid-level cadres as substitutes for internationally mobile health professionals in Africa. A desk review. Hum Resour Health.

[45] World Health Organization (WHO) (2007). Task shifting: rational redistribution of tasks among health workforce teams: global recommendations and guidelines.

[46] Brunie A, Wamala-Mucheri P, Otterness C, Akol A, Chen M, Bufumbo L (2014). Keeping community health workers in Uganda motivated: key challenges, facilitators, and preferred program inputs. Glob J Health Sci Pract.

[47] Akol A, Nalugya J, Nshemereirwe S, Babirye JN, Engebretsen IMS (2017). Does child and adolescent mental health in-service training result in equivalent knowledge gain among cadres of non-specialist health workers in Uganda? A pre-test post-test study. Int J Ment Health Syst.

[48] Nutbeam D, Harris E, Wise W. Theory in a nutshell: a practical guide to health promotion theories. New York: McGraw-Hill; 2010.

