# Supplementary material for: Qualitative evaluation of the outcomes of care and treatment for children and adolescents with nodding syndrome and other epilepsies in Uganda
Source: Infect Dis Poverty. 2019 Apr 30;8:30. doi: 10.1186/s40249-019-0540-x (PMC6489326; doi:10.1186/s40249-019-0540-x)
Supplement: Supplementary file 1 — Multilingual abstracts in the five official working languages of the United Nations (PDF 374 kb) [file 40249_2019_540_MOESM1_ESM.pdf]

Translation of the abstract into the five official working languages of the United Nations

التقييم النوعي لنتائج رعاية وعلاج الأطفال والمراهقون الذين يعانون من متلازمة الإيماء بالرأس وغيرها من أمراض الصرع في أوغندا.

كاثرين آبو، وآموس ديوجراتيوس مواكا، وبرنارد توليفا أوبار، وريتشارد إيدرو

مُلخّص

معلومات عامة: في عام 2012، أعلنت الحكومة الأوغندية انتشار وباء متلازمة الإيماء بالرأس (NS) في الضواحي الشمالية غولو، وكيتغوم، ولامو، وبادر. تمَّ وضع المبادئ التوجيهية للعلاج وإنشاء مراكز لعلاج متلازمة الإيماء بالرأس للتحكّم بالأعراض والتأهيل. إلّا أنَّه لا يزال هناك فجوة واسعة بين معايير الرعاية المُحدّدة مُسبقاً والرعاية الروتينية التي يتم تقديمها للمتضررين. تهدف هذه الدراسة إلى تقييم مدى الالتزام نوعياً بمعايير الرعاية الطبية المقبولة بالنسبة لمتلازمة الإيماء بالرأس؛ تحديد الفجوات فيما يتعلق برعاية الأطفال المصابين وتوفير الإشراف الطبي السريري (CSS) لموظفي الرعاية الصحية الأولية (PHC) في مراكز العلاج؛ وتحديد التحديات الصعوبات النفسية والإجتماعية التي يواجهها الأطفال المصابين ومقدّمي الرعاية لهم.

الطُرق: تمَّ إجراء دراسة الحالية الحالية في ضواحي غولو، وكيتجوم، ولامو، وبادر في أوغندا في الفترة الممتدة من سبتمبر إلى ديسمبر عام 2015. باستخدام نهج التدقيق السريري المكّون من خمس مراحل، تمَّ جمع البيانات عبر ملاحظاتٍ ومقابلاتٍ مباشرة تمَّ اجراؤها مع مقدّمي الرعاية الصحية الأولية الذين يعملون في المرافق الصحية العامة والخاصة غير الربحية، وكذلك مع مقدّمي الرعاية والقادة السياسيين. تمَّ تحليل البيانات النوعية باستخدام نموذج سيدل لمعالجة البيانات.

النتائج: أظهر التدقيق السريري والإشراف الطبي السريري، عدم الالتزام بالمبادئ التوجيهية للعلاج. لقد كان تدبّر متلازمة الإيماء بالرأس التي يعاني منها العديد من الأطفال دون المستوى، وهو ما أدّى إلى ضعف التحكّم بالأعراض وحدوث مضاعفات مثل الحروق الشديدة. إنَّ الأسباب الجذرية لهذه النتائج هي نفاد مخزونات الأدوية المضادة للصرع، وهجرة العاملين في القطّاع الصحي من مراكز عملهم فضلاً عن المسائل النفسية والإجتماعية. لم يكن هناك أي فريق متخصص متعدد التخصصات (MDT) لتأهيل المرضى وتمَّ استخدام نموذج تحويل المهام دون توفير إشرافٍ كافٍ، وهو ما أدى إلى فقدان المهارات التي تمَّ تعلّمها. وقد انضوت المسائل النفسية والإجتماعية والنفسية الجنسية المرتبطة بمتلازمة الإيماء بالرأس حالات الحمل المبكّرة، والعرض العلني للسلوكيات الجنسية والاعتداء على الأطفال.

الاستنتاجات: على الرغم من مشاركة أعضاء الفريق المتخصص متعدد التخصصات المعنيين في وضع المبادئ التوجيهية متعددة التخصصات لمتلازمة الإيماء بالرأس، لم يتم تطبيق الرعاية متعددة التخصصات في الواقع العملي. ثمّة حاجة ملحّة إلى مراجعة المبادئ التوجيهية السريرية لمتلازمة الإيماء بالرأس. ينبغي توفير إشرافٍ طبي سريري ربع سنوي والأدوية المضادّة للاختلاجات في المرافق الصحية للمجتمعات المتأثرة. كما ينبغي تنفيذ السياسات والبرامج القائمة للتعامل مع المسائل النفسية والإجتماعية والنفسية والجنسية التي تؤثّر على الأطفال الذين يعانون من متلازمة الإيماء بالرأس وغيرها من الأمراض المُزمنة.

Translated from English version into Arabic by Aalya Al-Beeshi, Revised by Shaima'a al-Fahel, through

TRANSLATORS
WITHOUT BORDERS

对乌干达罹患点头综合症和其他癫痫的儿童和青少年的护理及其疗效的定性评估

Catherine Abbo, Amos Deogratius Mwaka, Bernard Toliva Opar and Richard Idro

摘要

引言： 2012 年，乌干达政府宣布 Gulu、Kitgum、Lamwo 和 Pader 等北部地区暴发了点头综合征（NS）疫情。为疫情控制和患者康复，制定了相关指南和 NS 治疗中心。但是，为受影响者提供的常规护理质量仍远远低于预先确定的护理标准。本研究旨在定性评估该地区对 NS 公认临床护理标准的依从性; 发掘受影响患儿护理问题，并向治疗中心的初级卫生保健（PHC）工作人员提供临床支持监督（CSS）；并探索受影响患儿及其照顾者的心理状况。

方法：2015 年 9 月至 12 月，我们在乌干达 Gulu，Kitgum，Lamwo 和 Pader 地区开展了该案例研究。运用 5 阶段临床审计法，通过直接观察，并对采访在公共场所和私人非营利性医疗机构的的 PHC 提供者、护理人员和领导进行采访，采集了相关数据。最后使用 Seidel 模型处理分析了这些定性数据。

结果：临床审核和 CSS 显示该地区对治疗指南的依从性较差。许多受影响的患儿未能接受优质的 NS 控制，无法有效控制癫痫，引发了并发症，包括严重灼伤。造成上述情况的根本原因是抗癫痫药物频繁缺货，卫生工作者从工作站的流动以及社会心理问题。 几乎没有专业的多学科团队（MDT）提供服务以满足患者的复杂康复需求，并且采用了支持监督不足的任务转移模型，导致技能的丧失。 据报告，与 NS 相关的社会心理和性心理问题包括早孕，性行为暴露和虐待儿童。

结论：尽管 MDT 相关成员参与了多学科 NS 指南的制定，但在实践中并未实施多学科护理，因此迫切需要审查 NS 临床指南。 受影响社区的卫生机构需要季度 CSS 和持续抗惊厥药物供给。此外，需要持续实施现有的政策和方案，来处理影响 NS 和其他慢性病儿童的心理社会问题。

Translated from English version into Chinese by Qing-Yun Chen, edited by Jin Chen

CHINESE CENTER FOR DISEASE CONTROL AND PREVENTION
NATIONAL INSTITUTE OF PARASITIC DISEASES

Évaluation qualitative des résultats de soins et de traitement des enfants et des adolescents atteints du syndrome du hochement de tête et d'autres formes d'épilepsie en Ouganda.

Catherine Abbo, Amos Deogratius Mwaka, Bernard Toliva Opar et Richard Idro

Résumé

Contexte : En 2012, le gouvernement ougandais a déclaré l'état d'épidémie face au syndrome du hochement de tête dans les districts de Gulu, Kitgum, Lamwo et Pader, dans le nord du pays. Des directives de traitement ont été élaborées et des centres de traitement de cette maladie ont été créés afin d'assurer traitement symptomatique et réadaptation des patients. Il existait cependant un écart considérable entre les standards de soins définis à l'avance et la qualité de la prise en charge des patients. La présente étude a pour but d'évaluer qualitativement le respect des standards de soins cliniques reconnus pour le syndrome de hochement de tête, d'identifier les carences des soins dispensés aux enfants, d'offrir une supervision clinique et de soutien (SCS) au

personnel de soins primaires des centres de traitement et d'identifier les difficultés psychosociales rencontrées par les enfants affectés et les personnes qui en ont la charge.
Méthodes : cette étude de cas a été menée dans les districts de Gulu, Kitgum, Lamwo et Pader, en Ouganda, de septembre à décembre 2015. Suivant l'approche en cinq étapes de l'audit clinique, les données ont été recueillies dans le cadre d'observations directes et d'entretiens avec les personnels de soins primaires de centres de santé publics et de centres privés à but non lucratif, ainsi qu'avec des responsables d'enfants et des décideurs politiques. Les données qualitatives ont été analysées à l'aide du modèle de traitement des données de Seidel.
Résultats : L'audit clinique et la SCS ont révélé un respect médiocre des directives de traitement. Le syndrome du hochement de tête était souvent mal pris en charge, avec pour résultat un mauvais contrôle des crises et des complications allant jusqu'à des brûlures graves. Ces résultats étaient liés à de fréquentes ruptures des stocks de médicaments antiépileptiques, à la migration des professionnels de la santé depuis leurs lieux de travail et à des difficultés psychosociales. Il n'y avait quasiment aucune équipe pluridisciplinaire spécialisée (EPS) capable de répondre aux besoins complexes de réadaptation des patients et le modèle de transfert des tâches prévoyait une supervision en soutien inadéquate, avec pour conséquence la perte des compétences acquises. Les difficultés psychosociales et psychosexuelles rapportées en relation avec le syndrome du hochement de tête étaient des grossesses précoces, un exhibitionnisme des comportements sexuels et des abus sur des enfants.
Conclusions : malgré l'implication de membres importants de l'EPS dans l'élaboration de directives multidisciplinaires sur le syndrome du hochement de tête, il n'y a pas eu de prise en charge multidisciplinaire dans la pratique. Il est nécessaire et urgent de revoir les directives cliniques concernant cette pathologie. Des SCS trimestrielles et un approvisionnement continu en anticonvulsivants sont nécessaires dans les centres de soins des communautés affectées. Les politiques et programmes existants destinés à lutter contre les problèmes psychosociaux et psychosexuels des enfants atteints de syndrome du hochement de tête et d'autres pathologies chroniques doivent être mis en application.

Translated from English version into French by Suzanne Assenat, Revised by Eric Ragu, through

TRANSLATORS
WITHOUT BORDERS

Качественная оценка результатов ухода и лечения детей и подростков с кивательным синдромом и другими эпилепсиями в Уганде.

Кэтрин Аббо, Амос Деогратиус Мвака, Бернард Толива Опар и Ричард Идро

Аннотация

Общая информация: в 2012 году правительство Уганды объявило об эпидемии кивательного синдрома (КС) в северных районах Гулу, Китгум, Ламво и Падер. Были разработаны руководящие принципы лечения и созданы центры лечения КС для обеспечения симптоматического контроля и реабилитации. Однако по-прежнему существует большой разрыв между заранее определенными стандартами ухода и качеством обычной помощи, оказываемой пострадавшим. Данное исследование призвано качественно оценить соблюдение принятых стандартов клинической помощи при КС; выявить пробелы в уходе за больными детьми и предложить инспекцию клинической поддержки (ИКП) персоналу первичной медико-санитарной помощи (ПМСП) в лечебных центрах; и выявить психосоциальные проблемы, с которыми сталкиваются пострадавшие дети и их сиделки.

Методы: данное тематическое исследование проводилось в районах Гулу, Китгум, Ламво и Падер в Уганде с сентября по декабрь 2015 года. Используя пятиэтапный подход клинического аудита, данные собирались путем прямых наблюдений и интервью с поставщиками ПМСП, работающими в государственных и частных некоммерческих медицинских учреждениях, а также с сиделками и политическими лидерами. Качественные данные были проанализированы с использованием модели обработки данных Seidel.

Результаты: клинический аудит и ИКП выявили плохую приверженность рекомендациям по лечению. Многие пострадавшие дети получали неоптимальное лечение и поддержку при КС, что приводило к плохому контролю припадков и осложнениям, включая тяжелые ожоги. Первопричинами таких исходов были частые перебои с поставками противоэпилептических препаратов, миграция медицинских работников со своих рабочих мест и психосоциальные проблемы. На местах крайне редко работала специализированная мультидисциплинарная команда (МДК), которая могла предоставить комплекс услуг нуждающимся в реабилитации больным и использовалась модель перераспределения смен с неадекватной инспекцией поддержки, что приводило к потере выученных навыков. Согласно сообщениям о психосоциальных и психосексуальных проблемах, связанных с СК, они включают раннюю беременность, публичное проявление сексуального поведения и жестокое обращение с детьми.

Выводы: несмотря на участие соответствующих членов МДК в разработке междисциплинарных руководящих принципов по лечению КС, мультидисциплинарный уход на практике не осуществлялся. Необходимо в срочном порядке пересмотреть клинические рекомендации по лечению КС. Ежеквартальные ИКП и последовательное применение противосудорожных лекарств необходимы в медицинских учреждениях в пострадавших общинах. Необходимо внедрять существующие стратегии и программы по решению психосоциальных и психосексуальных проблем, затрагивающих детей с КС и другими хроническими заболеваниями.

Translated from English version into Russian by Michael Orlov, Revised by Alexander Somin,

through

Evaluación cualitativa de los resultados de la atención y el tratamiento de niños y adolescentes con síndrome del cabeceo y otros tipos de epilepsia en Uganda.

Catherine Abbo, Amos Deogratius Mwaka, Bernard Toliva Opar y Richard Idro

Resumen

Antecedentes: en 2012, el gobierno de Uganda declaró una epidemia de síndrome del cabeceo (NS, por sus siglas en inglés) en los distritos septentrionales de Gulu, Kitgum, Lamwo y Pader. Se formularon directrices de tratamiento y se establecieron centros de tratamiento del NS a fin de proporcionar control sintomático y rehabilitación. Sin embargo, aún seguía habiendo una gran diferencia entre las normas de atención preestablecidas y la calidad de la atención de rutina proporcionada a las personas afectadas. El objetivo de este estudio es evaluar cualitativamente el cumplimiento de las normas de atención clínica aceptadas para el NS, identificar brechas en la atención de los niños afectados, ofrecer supervisión de la asistencia clínica (CSS, por sus siglas en inglés) al personal de atención primaria (PHC, por sus siglas en inglés) de los centros de tratamiento e identificar los retos psicosociales a los que se enfrentan los niños afectados y sus cuidadores.

Métodos: este estudio se realizó en los distritos de Gulu, Kitgum, Lamwo y Pader en Uganda de septiembre a diciembre de 2015. Utilizando el enfoque en cinco etapas de auditoría clínica, se recopilaron datos a través de observaciones directas y entrevistas con profesionales de PHC que trabajan en centros de salud públicos y privados sin fines de lucro, así como con cuidadores y líderes políticos. Los datos cualitativos se analizaron utilizando el modelo de Seidel para el procesamiento de datos.

Resultados: la auditoría clínica y la CSS revelaron un cumplimiento deficiente de las directrices de tratamiento. Muchos niños afectados recibieron una gestión del NS que dista de ser óptima, lo que resultó en un control deficiente de las convulsiones y en complicaciones que incluyen quemaduras graves. Las causas fundamentales de estos resultados fueron la frecuente falta de existencias de medicamentos antiepilépticos, la migración de los trabajadores sanitarios de sus puestos de trabajo y problemas psicosociales. Apenas había un equipo especializado multidisciplinario (MDT, por sus siglas en inglés) para atender a las complejas necesidades de rehabilitación de los pacientes y se utilizó un modelo de rotación de tareas con una supervisión inadecuada de la asistencia, lo que condujo a una pérdida de habilidades aprendidas. Los problemas psicosociales y psicosexuales comunicados relacionados con el NS incluyeron embarazos precoces, muestras públicas de comportamientos sexuales y abuso infantil.

Conclusiones: a pesar de la participación de los miembros del MDT correspondientes en el desarrollo de directrices multidisciplinarias para el tratamiento del NS, la asistencia multidisciplinaria no se puso en práctica. Existe una necesidad urgente de revisar las directrices clínicas para el NS. Se necesitan CSS trimestral y medicamentos anticonvulsivos constantes en los centros de salud de las comunidades afectadas. Se requiere asimismo aplicar las políticas y programas existentes para tratar las cuestiones psicosociales y psicosexuales que afectan a los niños con NS y otras enfermedades crónicas.

Translated from English version into Spanish by Mayra León, Revised by Patricia Cassoni, through

TRANSLATORS
WITHOUT BORDERS
